# Coupled liquid biopsy and bioinformatics for pancreatic cancer early detection and precision prognostication

**DOI:** 10.1186/s12943-021-01309-7

**Published:** 2021-02-16

**Authors:** Jun Hou, XueTao Li, Ke-Ping Xie

**Affiliations:** 1 The South China University of Technology School of Medicine, 510006 Guangzhou, China; 2 The University of Texas MD Anderson Cancer Center Houston , Texas, USA

**Keywords:** Pancreatic cancer, Liquid biopsy, Circulome, Bioinformatics, Diagnosis, Prognosis

## Abstract

Early detection and diagnosis are the key to successful clinical management of pancreatic cancer and improve the patient outcome. However, due to the absence of early symptoms and the aggressiveness of pancreatic cancer, its 5-year survival rate remains below 5 %. Compared to tissue samples, liquid biopsies are of particular interest in clinical settings with respect to minimal invasiveness, repeated sampling, complete representation of the entire or multi-site tumor bulks. The potential of liquid biopsies in pancreatic cancer has been demonstrated by many studies which prove that liquid biopsies are able to detect early emergency of pancreatic cancer cells, residual disease, and recurrence. More interestingly, they show potential to delineate the heterogeneity, spatial and temporal, of pancreatic cancer. However, the performance of liquid biopsies for the diagnosis varies largely across different studies depending of the technique employed and also the type and stage of the tumor. One approach to improve the detect performance of liquid biopsies is to intensively inspect circulome and to define integrated biomarkers which simultaneously profile circulating tumor cells and DNA, extracellular vesicles, and circulating DNA, or cell free DNA and proteins. Moreover, the diagnostic validity and accuracy of liquid biopsies still need to be comprehensively demonstrated and validated.

## Introduction

Pancreatic ductal adenocarcinoma (PDA) is one of the most aggressive cancers and currently the third and seventh leading cause of cancer-related death in the United States and China, respectively [1, 2]. PDA at early stage has nonspecific symptoms, which is one of the most important reasons for a low 5-year survival rate in PDA [3]. While for late stage PDAs, accounting for the majority of cases, a pathological confirmation is required with tissue biopsies obtained from risky procedures and could be inconclusive or ambiguous in up to 20 % of cases due to scarcity of tumor cells in the biopsies [4]. In addition, PDA is also characterized with the lack of sensitive and specific biomarkers for early detection and preventive screening, no efficient and targeted therapeutics, prone to develop intrinsic and acquired chemoresistance [5]. Currently, the only curative treatment for PDA is surgical removal, however, which is only possible for a small proportion of PDA patients. Over 80 % of PDA patients are diagnosed at a late stage with distant metastases presented when only adjuvant therapy is feasible.

Early diagnosis and detection of recurrence or metastasis can greatly improve patient outcome. To date, the only diagnostic biomarker for PDA is serum CA19-9 level, which is neither diagnostic nor specific. High CA19-9 level is most often detected in advanced PDA, but less commonly found in early stage PDA. Moreover, an elevated CA19-9 level is also be detected in various benign and malignant conditions, including pancreatitis, cholestasis, gastric cancer, etc. Additionally, the use of CA19-9 as a PDA biomarker is by the lack of expression of CA19-9 in about 10 % of the Caucasian population. At the same time, imaging detection, such as contrast-enhanced computed tomography (CT), magnetic resonance imaging (MRI), and endoscopic ultrasonography (EUS) are insufficient for the early detection of PDA due to the screening limitations.

Ideally, we could obtain non-invasive, reliable, and reproducible biomarkers with clinical potential for cancer early diagnosis and patient outcome prediction. Liquid biopsies, such as circulating tumor DNA (ctDNA), circulating tumor cells (CTCs), extracellular vesicles (EVs), plasma proteomics and circulating tumor cells (CTCs) hold great promise to be used as such real-time and remote tools. Unlike other solid tumors especially lung cancer and breast cancer where a few circulating biomarkers have entered clinical practice, very limited blood-derived biomarkers are under evaluation for PDA diagnosis or monitoring, except for CA19-9, largely underdeveloped compared to other tumors. One example is the CellSearch system-based diagnostics utilizing EpCAM and cytokeratin expression on isolated epithelial cells, an FDA cleared diagnostics for metastatic breast, colon, and prostate cancer, which was evaluated for the diagnosis of PDA and achieved accuracies ranging 11 ~ 78.5 %, indicating great variation in detection rate for PDA [6]. Other molecular alterations evaluated to diagnose PDA include KRAS mutations in CTCs, miRNAs in cancer EVs, and heparan sulfate proteoglycan glypican 1 (GPC1) in extracellular vesicles. Unfortunately, the sensitivities and predictive performance of these circulating markers demonstrated great discrepancies between different studies, even more striking between tumor and CTC status. As one study revealed that 97 % of tumors carried mutant KRAS, 18 % of the CTCs were found to carry only the KRAS wild type allele, even those from metastatic tumors [7]. Thus, it is possible that employing single biomarkers in liquid biopsy might capture only partial tumor biological features due to inherited limitations linked with CTC enrichment and identification, ctDNA isolation etc., leading to a low consistency and false negativity of the results.

To improve detection rate of liquid biopsies, diverse strategies have been developed. One of these is to combine different types of liquid biopsies to capture more possible biological features of primary and metastatic tumors. Following this concept, more and more studies on screening diagnostic and prognostic markers focus on the circulome, instead of single molecules in liquid biopsies. The ‘circulome’ is defined as a collection of circulating molecules, cells, factors, proteins and other macromolecules. In practice, the usage of ‘circulome’ and ‘liquid biopsy’ largely overlapped. Here in this review, we tend to make a clear distinguish between these two terms. The definition and usage of ‘circulome’ will be restricted to referring the information and biomarker sets derived from a combination of multiple types of liquid biopsies. The circulome makes usage of complementary nature of different liquid biopsies and is deemed to overperform single type liquid biopsies in terms of profiling more cellular or molecular compartments shed from tumor tissues.

## Tumor circulome

### Circulating tumor‐derived proteins

The concentration of blood proteins has been historically used for tumor screening, diagnosis, and prognostic monitoring. Well-established circulating tumor markers include the prostate-specific antigen (PSA) for prostate cancer screening [8] and carbohydrate antigen 19 − 9 (CA19-9) for postoperative follow-up of pancreatic cancer recurrence [9]. In general, these blood proteins tend to be less frequently used for monitoring purposes due to their long half-lives [10]. In addition, with the development of novel biomarkers of high specificity and accuracy, including markers in liquid biopsies, the clinical significance of these circulating protein markers is dissipating. Until recently, the advancements in high throughput proteomics platforms, such as Matrix-assisted laser desorption/ionization time-of-flight mass spectrometry (MALDI-TOF MS), have enabled the simultaneous measurement of panels of proteins. While the combination of multiple circulating protein markers is anticipated to improve the false positives and false negatives [11, 12].

In PDA, the diagnostic value of multiple blood proteins has been evaluated in comparison to CA19-9. The expression levels of circulating tissue metalloproteinase inhibitor-1 (TIMP1) and Leucine-rich alpha-2-glycoprotein 1 (LRG1), thrombospondin-2 (THBS2) showed comparable diagnostic performance to CA19-9. While the diagnosis of early PDA could be significantly improved by combining the expression of these blood molecules with CA19-9 [13, 14]. It is worth to mention here, similar to circulating proteins, blood metabolites demonstrated diagnostic potentials to detect PDA at early stage, either alone or in combination with CA19-9 or other proteins, as exemplified by recent studies which revealed that plasma metabolites, such as acetylspermidine, diacetylspermine, indole-derivatives, and lysophosphatidylcholines could distinguish PDA from healthy subjects and benign pancreatic disorders [15, 16].

### Circulating tumor DNA (ctDNA)

Circulating tumor DNA are deemed to be secreted from the tumor, either primary or metastatic, or CTC by the mechanism of cell necrosis, lysis, or apoptosis [17]. Dependent on the cell origin, a fraction of ctDNA is circulating cfDNA originating from cancer cells. The size of ctDNA is mainly around 160 base pairs, such as ctDNA fragments associated with nucleosomes [18]. However, longer double-strand fragments (> 10 kb) encapsulated within EVs have been also designated as ctDNA, which carry tumor genomic information and can be used to identify relevant mutations in patients with pancreatic cancer [19]. Nevertheless, as widely accepted, ctDNAs are short genomic fragments spanning all chromosomes.

ctDNAs present as valuable clinical biomarkers with two potential applications in oncology for therapeutic decision making and early detection of relapse. It is confirmed in several cancers that the quantity of ctDNA is correlated with tumor burden, for instance 47 % of patients with early stage cancers of any type had detectable ctDNA, whereas the fraction of patients with detectable ctDNA was 82 % for patients with advanced cancers [20]. Qualitative information of tumor genome, such as mutations, amplifications, deletions and translocations, can also be retrieved from profiling ctDNA, which allows the identification of genetic alterations for patient stratification or therapeutic response prediction. A milestone of liquid biopsy was reached by cobas® EGFR Mutation Test V2 (Roche Diagnostics), the first ctDNA-based companion diagnostic test approved by the FDA [21]. This test is used to guide the use of epidermal growth factor receptor (EGFR)-tyrosine kinase inhibitors on the basis of specific EGFR-sensitizing mutations in patients with non-small cell lung cancer (NSCLC). Recently more similar ctDNA tests in other types of cancer have been approved by the FDA or validating in clinical settings, such as Epi proColon^®^ for colorectal cancer screening and Signatera test to identify molecular residual disease and recurrence in multiple types of solid tumors [22–24]. Based on the observations obtained from clinical trials, ctDNA is appreciated as a promising noninvasive biomarker to assist patient selection.

Moreover, ctDNA of the same patients was sampled consecutively before and after surgery in breast cancer, certain somatic mutations such as PIK3CA c.3140A > T (p.H1047L) were undetectable in subjects achieved disease-free and detectable in subjects presenting minimal residual disease. Interestingly, the abundance of this mutation showed a remarkable increase 1.9 months before clinical relapse was diagnosed [25]. Similarly, in colon cancer an association between ctDNA post-operative positivity and relapse-free survival was observed. And the reappearance of blood ctDNA in subjects, which initially acquired ctDNA negativity, was predictive for disease relapse [26]. Remarkably, the detection of ctDNA is up to 10 months earlier than the detection of circulating tumor cells and circulating protein markers, resulting in an exceptional lead time to clinical diagnosis of tumor relapse [20, 26]. These observations and others in multiple types of cancers demonstrated that the potential of ctDNA to monitor therapeutic efficacy, to track mutational evolution spectrum under therapy-induced selective pressure, and to detect the recurrence of cancer at early stage.

In the specific context of the PDA, numerous studies have already been conducted to screen for PDA diagnostic biomarkers in ctDNA. Most of these studies utilized KRAS mutations to target ctDNA. However, the sensitivities ranged between 27 % and 81 %, and specificity between 33 % and 100 % [27–30], requiring more sensitive and specific strategies for early PDA identification. Yet the concordance of the detected KRAS mutation between primary tumor and ctDNA is unfavorable on the other hand, varying from 25–75 % [31]. Nevertheless, a correlation between the presence of KRAS mutation in ctDNA and poor prognosis of PDA patients was established in some studies [27, 32]. Besides gene mutation, DNA methylation is an emerged diagnostics in liquid biopsy. Shen et al. showed that the methylation patterns in ctDNA can detect PDA, even tumors at early stage [33].

### Circulating tumor cells (CTCs)

CTCs are a population of tumor cells with low abundance in the blood stream. The average concentration of CTCs is 10 ~ 100 in 10^6–8^ white blood cells, depending on the method of CTC enrichment. Compared to other liquid biopsies, CTCs have greater potential as quantitative tumor biomarkers for early diagnosis, MRD monitoring, therapeutic response and prognosis prediction, especially for solid tumors.

These cells are believed to be detached from the primary tumor and be the main source of metastases [34, 35]. Given the nature of shedding from the primary tumor, CTC pool in peripheral blood might comprise tumor cells detached from different regions of the same tumor, or from multiple loci, or even from both primary tumor and (occult) metastasis. In this way, CTCs recapitulate better the whole-body burden of tumor than tissue biopsies obtained from a single spot. It can also be speculated that inspecting CTCs can get better insights into tumor heterogeneity, both spatial heterogeneity and temporal heterogeneity. Moreover, unlike ctDNA, CTCs can provide enormous information, not only genetic variation but the expression of genes and cytoplasm proteins, of cellular contents which are preserved and garnered by the cell membrane. Thus, it is possible to integrate multiple-level information obtained from CTCs to get a comprehensive landscape of tumor heterogeneity and evolution, as well as molecular distinctions. In addition, CTCs also allow for ex vivo culture, which gives the chance to perform personalized therapeutic response prediction and drug screening [36, 37].

Conventionally, the detection of CTC linked to PDA is based on the methodologies including density centrifugation and RT-PCR detection of tumor markers CEA [38], cytokeratin 20 [39], or EpCAM [40]. By using a such system, which captures tumor cells from whole blood based on EpCAM and cytokeratin expression, a PDA diagnosis was made in 32 % of patients in a cohort consisting of advanced PDA and 7 % in a cohort consisting of early stage PDA [41, 42]. When applying alternative strategies for CTC detection, the detection rate of cell size-based method could be reached to 93 % in advanced PDA [43], and 67 % with cytomorphology-based method [44]. Recent studies explored the strategy of identifying CTC by targeting KRAS mutation. However, this approach showed high discrepancy between tumor and CTC. A study enrolled patients with various pancreatic disorders, including PDA and non-malignant diseases, and detected mutant KRAS in five out of twelve metastatic PDA, compared to a detection rate of 97 % in tumor samples [7].

### Extracellular vesicles (EVs)

These membrane-derived small bodies are lipid bilayer-delimited particles encompassing diversified biological content released from cells. Virtually, many subcellular compartments can generate and all cell types can release EVs, therefore, the composition of EVs is able to reflect the ongoing cellular activities and pathological processes in the cells from which they originate. Another consequence is the content and cargo load of EVs can be largely heterogeneous and nucleic acids, proteins, lipids, and metabolites all can be packed within a vesicle, depending on the type and state of their parent cells.

The main functions of EVs are linked to their capacity of carrying and transferring a cargo. Previous studies have proven that EVs have important roles in intercellular and interorganismal communication and regulating physiological and pathological processes by transporting free RNAs, lipids, or proteins [45–48]. Li and others have concluded that EVs can also facilitate the migration of membrane-bound receptors and antigen presentation complexes, implying unique roles in immune response and regulation possessed by EVs [49, 50].

Profiling the content of tumor-derived EVs might be able to identify the origin of cancer, distinct genomic traits, and metabolic status of cancer [51]. Interestingly, some studies observed distinct RNA and protein profiles of EVs from their parent cells, indicating an active and selective loading mechanism employed by EV vehicle [52–54].

It has been shown that macrophage inhibitory factor (MIF) was detected in pancreatic cancer derived EVs, which can be uptaken by Kupffer cells and in turn promoted a series of molecular and cellular processes in Kupffer cells as well as cells at distance, including TGF-β (transforming growth factor beta) secretion by Kupffer cells, and fibronectin secretion by neighboring hepatic stellate cells. The effects of the activation of these processes were believed to trigger pancreatic metastases [55]. The diagnostic and prognostic value of the content in EV in PDA has been evaluated in numerous studies, and the targeted molecules included miRNAs, genes, and proteins. GPC1 enriched in PDA-derived EVs was detectable in the serum of PDA patients with high specificity and sensitivity, even at early stage PDA [56]. Similarly, the expression level of signature miRNAs in EV can be used to make an early diagnosis of PDA [57]. However, the sensitivity of EV signatures ranged from 39–100 %, and their correlation with PDA prognosis varied greatly between different studies [56–59].

### Circulating tumor RNA (ctRNA) and other circulating biomarkers

More extractable liquid biopsies from peripheral blood also include ctRNAs, TEPs, miRNAs, and metabolites. Different analytes carry different information about the tumor genome. For instance, from ctRNA the expression information about circulating gene transcripts and non-coding RNAs can be obtained. While RNA expression profiles extracted from TEPs has been shown to discriminate tumors from healthy tissues, and further make molecular subclassifications in multiple cancers [60]. The global changes induced by the development of a tumor also include metabolic processes, thus the level of circulating metabolites or end metabolites in urine might be able to reflect the molecular and cellular alterations in tumor cells, as evidenced by disturbed metabolic regulation in pancreatic cancer and lung cancer indicated by the aberrant plasma levels of BCAAs [61–63].

Recent years excreted body fluids have also been investigated to determine whether they are promising non-invasive tumor biomarkers. Specifically, in PDA it has been reported that driver mutations such as KRAS G12V and G12D mutations are detected in DNA purified from pancreatic juice when pancreatic duct biopsy and pancreatic juice cytology are performed with no evidence of malignancy. The genetic analysis and pathological test using a resected specimen verified the diagnosis of early primary PDA harboring KRAS G12V mutation. Additional test has revealed that other premalignant lesions or in situ carcinoma are KRAS G12D mutation positive [64].

### Circulome: integrated liquid biopsies

As described above, discordant detection results of single biomarkers cast a shadow over the future application of liquid biopsy, while combined liquid biopsies can substantially improve cancer diagnosis and prognosis prediction [65]. Accordingly, the concept of ‘circulome’ referring to integrating multiple biomarkers in liquid biopsy was developed to improve the sensitivity of cancer detection. The biomarkers to be integrated could be DNA, RNA, protein, and other molecules in liquid biopsy. One example showed that the joint performance of two EV-derived proteins (GPC1 and CD63) in distinguishing PDA from healthy subjects can reach up to a sensitivity of 99 % and specificity of 82 % [66]. This proof-of-concept was further applied to predict the outcome of cancer patients, including PDA patients, through a probability model built with genetic alterations and protein biomarkers in liquid biopsies [67]. More interestingly, the circulome strategy not only significantly improved the sensitivity of earlier cancer detection, but also showed capacity to localize the original organs of the profiled cancers [67]. This concept has great potential to expanded to other liquid biomarkers, such as metabolites, methylated DNA, and molecules in EVs. For instance, methylation is a robust molecular character and already abundant in the early tumorigenesis. In this sense, the combined detection of methylation and another form of liquid biopsy with a low abundancy could be evaluated as a circulomic signature for early cancer detection.

**Fig. 1:**
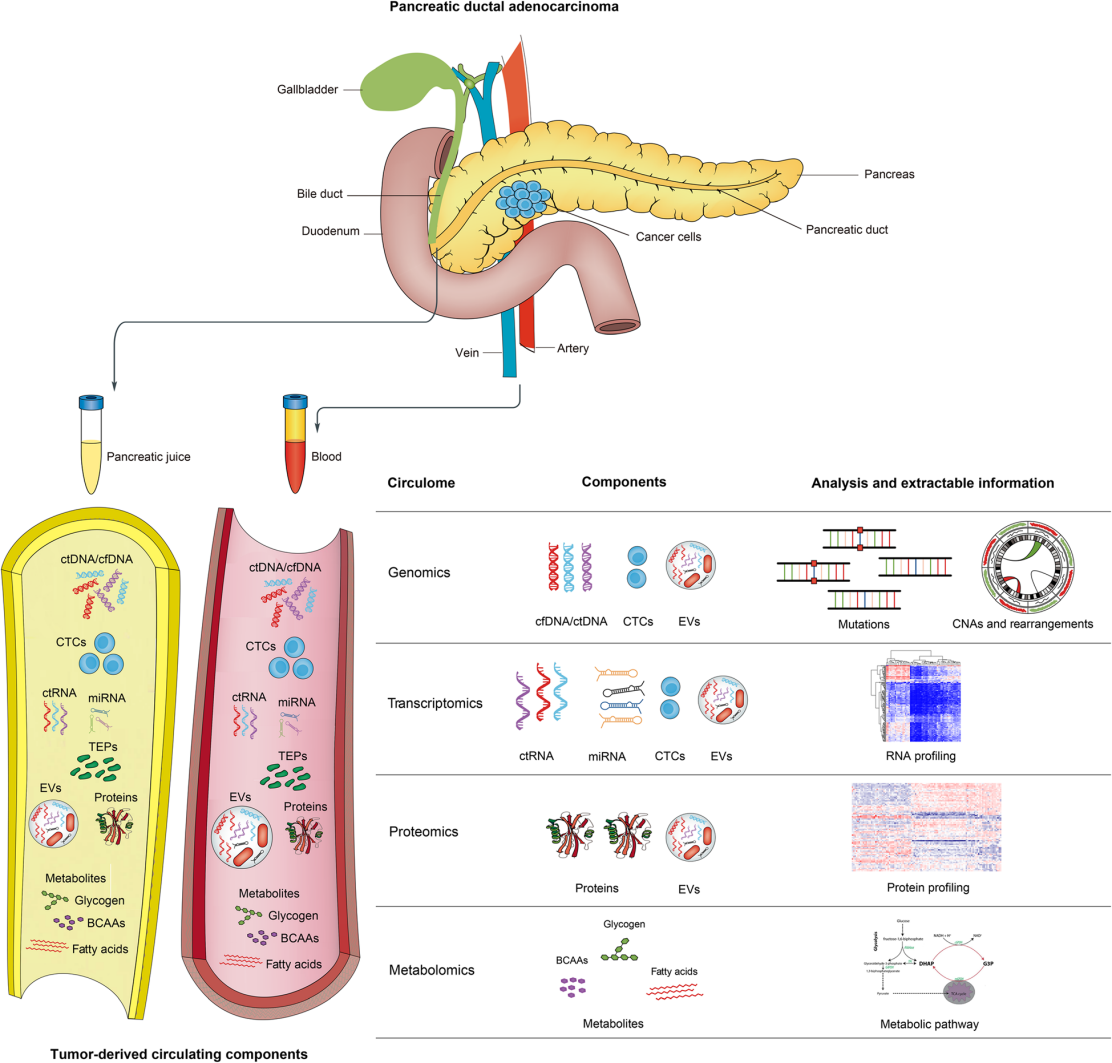
Integrated strategies for detection of tumor from liquid biopsies. Various tumor-derived circulating components can be used as a source of liquid biopsies, including circulating cell-free DNA (cfDNA), circulating tumor DNA (ctDNA), circulating tumor cells (CTCs), circulating tumor RNA (ctRNA), circulating miRNA, tumor-educated platelets (TEPs), extracellular vesicles (EVs), circulating tumor-derived proteins, and circulating metabolites. Each element can extract one or more levels of information about the genome, the transcriptome, the proteome or the metabolome.

Integrating liquid biopsies with distinct sources is highly possible the most powerful tool to reliably diagnose and monitor cancer, to understand cancer heterogeneity, and to decode the tissue and cell origin of cancer by providing comprehensive and complementary information about tumor biology. Moreover, collective information derived from multiple liquid biopsies creates possibility to dynamically track genomic and biomolecular alterations continuously developed during tumor progression and evolution (Fig. 1).

## Tissue and cell origin of liquid biopsies

To extract proper information for diagnostic, predictive, and monitoring purpose, the utmost important issue is to enrich sufficient liquid biopsies as well as select tumor-specific ones with right tissue and cell origin (for EVs). Although cancer liquid biopsies have been considerably profiled, the biology of liquid biopsies in healthy or noncancerous conditions, such as aging and chronic inflammation, have not been fully understood. The knowledge of the normal composition of plasma liquid biopsies and the contribution of different tissues to plasma pool of liquid biopsies is decisive to develop the tissue-specific isolation strategies and to trace the tissue origin of liquid biopsy components in plasma.

Currently the cell origin of EVs is usually not estimated. The isolation of EVs of any resource is uniformly based on the surface markers, such as CD63 and CD81. cfDNA encounters the same circumstance, where no cell origin is regularly assessed for blood-isolated cfDNA. For CTCs, the conventional sorting technique can distinguish limited cell types in a single experiment [68]. With the latest development of CyTOF, maximal 40 parameters can be processed to categorize the studied cells. Moreover, these techniques mainly utilize antibodies to detect and measure cells, while the available antibodies are limited. On the other hand, the expression of cell surface proteins is highly regulated and dynamic, and readily adapted to diversified conditions and stress like oncogenesis. In this sense, classical cell surface markers, which are established mostly in healthy cells and normal physiological conditions, are not adequate to classify liquid biopsies when their parent cells are cancerous, especially when tissue origin is considered simultaneously.

Some studies have proven that the abundance of ctDNA is correlated with its tissue origin, stage of tumor development, and tumor burden. The ctDNA was detectable in > 75 % of patients with advanced pancreatic, ovarian, colorectal, bladder, gastroesophageal, breast, melanoma, hepatocellular, and head and neck cancers, but in less than 50 % of primary brain, renal, prostate, or thyroid cancers. In patients with localized tumors, ctDNA was detected in 73, 57, 48, and 50 % of patients with colorectal cancer, gastroesophageal cancer, pancreatic cancer, and breast adenocarcinoma, respectively [20]. Some studies argued that ctDNA is unlikely to be caught from patients with tumors of a small tumor burden, for example at early or asymptomatic stage [69, 70].

In addition, the cell origin of liquid biopsies might determine the composition and biology of liquid biopsies. For example, ctDNA is believed to be derived from apoptotic cells and consequently have short length. Hence, uneven distribution of genome fraction is a feature of apoptotic cell-originated ctDNA. The apoptotic nature results in the difficulties in enriching ctDNA fragments from plasma to obtain unbiased information of tumor genome and to gain stable sensitivity and specificity of ctDNA-derived biomarkers. Moreover, the discordant genotypes between tumor biopsy and blood-based analytes might partially result from targeting incomplete genome carried by ctDNA [71].

Establishing specific protocols for different liquid biopsies to standardize specimen collection, processing, and laboratory procedures could definitely increase the accordance between different studies, and more importantly could help to decode the tissue and cell origin of liquid biopsies. Incorporating the information on where and how liquid biopsies are released into the circulation will not only improve the compatibility across studies, but deepen the understanding of circulome biology in liquid biopsies. Another plausible solution is to develop label-free in silico algorithms which can deconvolute the cell origin of liquid biopsies based on genomic data, gene expression data, or proteomic data. Such computational strategies have been widely applied to determine the relative abundance of immune cells in tumor microenvironment with bulk tumor expression data [72, 73]. Exploring the cell origin of liquid biopsies by such strategies has been exemplified by Hoshino and his colleagues. By using high throughput mass spectrometry and bioinformatics analysis, they demonstrated that the uptake of EV presented cell and organ preference, which was probably determined by EV cell origin and mediated by the expression of exosomal integrins [74].

PDA is asymptomatic at early stage, in addition, due to the anatomical position of the pancreas and the cannulation of stromal cells in tumor mass, PDA is very difficult to be detected and distinguished from benign pancreatic disorders at early stage. The facts that limited number of liquid biopsies can be obtained from early PDA – one of the lowest among various cancers [75], and the majority is healthy or stromal cells confer extra importance in PDA on figuring out the tissue and cell origin of liquid biopsy, especially ctDNA and EVs, which are already released during early tumorigenesis. With selected tumor specific liquid biopsies, additional enrichment procedures could be applied in order to gain sufficient material for tumor detection and assessment. Moreover, the simultaneous enrichment of both tumor-derived ctDNA and EVs might provide significantly higher detection rate considering that both EVs and ctDNA can be released from all composites of cancer niches to trigger tumor growth.

A unique feature of pancreatic cancer is the direct contact of tumor cells with exocrine and endocrine system of pancreases. As a result, various tumor associated materials, such as CTCs, ctDNA, ctRNA, and EVs can be released into pancreatic juice, making it an ideal repertoire for sampling integrated liquid biopsies of PDA [76–79]. These composites in pancreatic juice contain genetic, genomic, as well as proteomic information and could be utilized to detect the most common biomarkers for diagnosing or monitoring PDA. Molecular profiling of pancreatic juice is extraordinary appreciated when negative results are obtained from pathological or cytological examination.

## Profiling the most proper analytes to understand cancer heterogeneity

Each component of circulome holds unique prospect for being tumor biomarker. The understanding of their distinct superiority can lead liquid biopsies to more powerful tools for cancer diagnosis, monitoring, and therapeutic decision making. The half-life of ctDNA is quite short (~ 2.5 h) therefore providing ideal real-time snapshot of tumor cells with respect to treatment response evaluation and dynamic tumor status assessments in various pathophysiological conditions. While ctDNA is not appreciated for tracking the evolutionary trajectory due to its drawback of low abundance. In most circumstance, circulome, instead of single type of liquid biopsy, can be employed to capture more facets of tumor genome and to improve the sensitivity and accuracy of the prediction. The above-mentioned examples in solid tumors demonstrated that the integration of liquid biopsies or circulome can largely enhance cancer surveillance. CPs have a great amount in peripheral blood, which can compensate for the limited abundance of ctDNA. In pancreatic cancers the diagnosis made by the combined KRAS mutations in ctDNA and CA19-9 is more sensitive than the KRAS mutation in ctDNA alone [30]. Impressively, an FDA approved multianalyte blood test integrated CPs and ctDNA profiles yielded satisfactory sensitivity ranging from 69–98 % for the detection of five cancer types (ovary, liver, stomach, pancreas, and esophagus) while maintaining a high specificity of 99 % [67].

Tumor heterogeneity is believed to be the source of drug resistance. Therefore, deciphering the heterogeneity of tumors holds promise to clarify the mechanism of drug resistance. While the heterogeneity is hard to be captured by the conventional sampling approaches. Profiling circulome consisting of multiple types of liquid biopsies might capture the full heterogeneity of tumors. It has been demonstrated that integrating exosomal RNAs (exoRNAs) with ctDNA surpassed ctDNA alone in detecting EGFR mutations in NSCLC [44]. It has been accepted that ctDNA is shed by necrotic or apoptotic cells while exoRNA is shed by living cells, the combination of these two components would facilitate the capture of the full tumor heterogeneity, reflecting different spatial origin and molecular cellular aspects of tumor biology, and increase the sensitivity of mutation detection in plasma or serum. It has been also verified that ctDNA-exoRNA integration increased the sensitivity of EGFR mutation detection in plasma in NSCLC patients without distant metastasis [44, 80, 81].

PDA is a highly heterogeneous and molecularly complex cancer with significant differences observed in patient outcome. Currently, histopathology-based subtyping is poorly correlated with PDA prognosis and individual response to the treatment. Recently, a study concluded that the total number of CTC might have prognostic impact on PDA patients. PDA patients with > 3 CTC/ml achieved a worse overall survival (OS) than patients with 0.3–3 CTC/ml [75]. Interestingly, metabolic rewiring was proposed to subclassify PDA based on a study on PDA cell lines and metabolic subtypes showed possible correlation with response to metabolism-based drugs [82]. Three PDA subtypes were proposed including “glycolytic”, with elevated glycolysis and serine pathways; “lipogenic”, with lipid and electron transport chain metabolite enrichment and high lipogenesis gene expression; and “slow proliferating” PDAs low in amino acids and carbohydrates. These subtypes were shown to have different responses to various metabolism-based inhibitors. Whether these metabolic subtypes have counterparts in primary PDA are well worth further research to validate.

Lack of sensitive and specific biomarkers is one of the most important challenges for PDA early detection and preventive screening. New-onset diabetes mellitus (nDM), is a recognized paraneoplastic condition preceding PDA diagnosis [83]. The association between the duration of DM and the risk of PDA has been substantially studied [84, 85] and the increased incidence of PDA was identified only in the nDM patients over the age of 50 years (DM of < 3 years duration), with a 6–8-fold higher 3-year risk compared to general population [86, 87]. Distinguishing DM of type 2 in elderly patients from nDM caused by PDA has important implications in the field of early detection of PDA, providing an opportunity of curative therapy to this group of patients.

Chari et al. demonstrated a strategy for early detection of resectable PDA in this high risk group by showing that approximately 1 % of nDM were diagnosed with PDA in 3-year period [39]. Some studies developed clinical models to diagnose PDA in nDM populations, which usually included age, change in blood glucose, and weight loss as the model parameters [88, 89]. Although these models showed encouraging preliminary results in distinguishing manifesting nDM of PDAC from other new-onset DM, the sensitivity and specificity of these models usually were below 80 %. The utility of biomarkers is speculated to assist the diagnosis of PDA in asymptomatic nDM subjects, as demonstrated by a study using serum or plasma CA19-9 as the adjuvant biomarker [90]. Perspectively, we could speculate that more specific biomarkers, such as miRNAs, mRNA, ctDNA, and molecules in EVs, especially the combination of these biomarkers could improve largely the early diagnosis of PDA in nDM populations, even in pre-diagnostic phase.

## Overcome the challenges imposed on circulome

The rarity in blood (e.g.10 CTC in a background of 10E + 6 white blood cells and 10E + 9 red blood cells) is the inherited drawback of most types of liquid biopsies, especially CTC. Currently, most CTC-enrichment methodologies rely on the surface marker-based epithelial cell capture. There is an abundance of data in breast, colon, and prostate cancer demonstrating that the presence of these circulating epithelial cells is associated with more aggressive disease [91]. However, the limitation of epithelial capture-based approaches rooted from the reliance on the expression of epithelial markers on cell surface. In many cases, the down-regulation, even loss, of these surface marker or epithelial-mesenchymal transition exists in tumor cells, making reliably enriching real CTCs a challenge. Other strategies are needed to guarantee a successful collection of CTCs. Another challenge to CTC is to decipher the spatial origin of CTC, shed from the primary tumor or metastatic tumor, from which part of the tumor.

Isolation and enrichment of CTC of PDA is particularly challenging because PDA has been defined as a cancer with one of the lowest number of CTCs in circulation [75]. And as described above, the CTC capture and enrichment in PDA is based on the expression CEA, CK20, or EpCAM. The expression of these markers is de-regulated in malignant cells, and correlated with the aggressiveness and histopathology of PDA, making this strategy less reliable. In addition to optimize the isolation and enrichment methodology, we could speculate that pancreatic juice might be a better source to attain sufficient CTC because of high concentration of tumor cells released from primary PDA. Indeed, it has been verified for long time that mutated KRAS and TP53 are more often detected in pancreatic juice than in other body liquid, such as plasma [92].

As for EVs, although a wealth of information about the functional status of the parent cells can be retrieved from EV’s repertoire, the utility of EVs in oncology is yet full of challenges. First, almost all types of cells can generate and release EVs. Under this circumstance, how to select EVs originated from tumor cells is an unsolved question. Moreover, cells release a substantial number of EVs per day. A high level of heterogeneity across EVs is existed in EV’s size, membrane composition and markers, and contents. Thus, understanding how exactly proteins and nucleoid acids are selected and loaded into EVs and how trafficking is regulated will be crucial for filtering out informative EVs from EV pool for oncological applications. The necessity was illustrated by recent studies where the mutant KRAS in EVs could be detected in a proportion of healthy individuals, patients with non-malignant pancreatic disorders, and 7.4–25 % of PDA patients [58, 59], indicating spontaneous somatic mutations encompassed in EV cargo and disappointing diagnostic performance by unfiltered EVs.

## Bioinformatics challenges and perspectives in cancer circulome

In the past decades, many studies of liquid biopsies have been published. However, there is low concordance between different studies. The inconsistencies might have derived from the utility of different sample preparation or high throughput platforms, but might also from biological divergence borne by the sampled liquid biopsies [93], Similarly, the sensitivity and specificity of circulomic biomarkers were found highly inconstant across different studies. Bioinformatic methodologies strengthened greatly the research and application of liquid biomarkers, however, there are limitations in analysis yet to be improved.

The low abundance is a common shortcoming of liquid biopsies. The low number of available target molecules in liquid biopsies dictates the poor capability to detect genomic variations with low frequency. Taking the primary tumor burden of 10 cm^3^ as an example, the resulting plasma VAF of ctDNA corresponds to 0.1 %, which means on average just six molecules per tube blood (10 ml) carrying the respective mutation can be sampled [94]. This fact exposes considerable challenges to sequencing as well as analytic approaches of genomic variations. Sequencing of a panel of genes, instead of the whole genome, could ensure a high degree of sensitivity on the target genes by sequencing each nucleotide of interest thousands of times. However, the increase of sequencing sensitivity in turn leads to higher false positivity which requires advanced bioinformatics or deep machine learning algorithms to eliminate high background noise in sequencing data and to diminish the false positive results. In addition, new analytic strategies need to be developed to address these challenges. For instance, the technique and analysis employing unique molecular identifier (UMI) can be adopted for the purpose of studying liquid biopsies [95], which employs unique tags to facilitate bioinformatic alignment of sequences derived and enables errors to be easily identified and excluded from subsequent analyses.

Another challenge to bioinformatics for tumor circulome is to differentiate tumor mutations from background somatic mutations. In a healthy person, somatic mutations exist with a rate of 2 ~ 6 mutations per 1 Mb [96], indicating most of the mutations called in liquid biopsies might be relevant to only normal biological functions. Extensive and comprehensive studies should be conducted to establish the mutation spectrum of liquid biopsies and blood cells in both healthy individuals and PDA patients.

Different omics data of liquid biopsies is suspected to complement each other for a complete picture of tumor genome. The methylation traits are one of the most studied and used cell specific marks in tumor biology because genome-wide methylation patterns are distinguished between different cells including between normal and malignant cells. A newly developed profile, nucleosome occupancy patterns, is suspected to align with the cell specificity. Different cells can have different nucleosome organizational features [97, 98]. The occupancy of nucleosomes over regulatory regions can be obtained from WGS data. The presence or absence of a nucleosome over a regulatory region dictates whether transcription factors are binding to the specific regions or not. While the specific occupancy at given positions is found in some cells but not others. And moreover, to quantify nucleosome occupancy, it is essential to obtain the information about the percentage of cells that contain a nucleosome at a given position, in addition to the position information of nucleosomes.

## Future directions

It is well accepted that EVs are released from viable cells, while other liquid biopsies are derived from apoptotic cells or dying cells. However, the most important nature of tumor cells is uncontrolled proliferation. Thus, the aggressive and highly proliferative cancer cells contribute more to the tumor biology than cancer cells undergoing apoptosis. The level of cellular and molecular heterogeneity in blood is not less than that in tumor microenvironment. Various types of cells, cytokines, and molecules could be found in blood. Given its circulating and accessible attributes, liquid biopsies compose a repertoire of cells or molecules derived from all cell types located at any part of the body. Also, the kinetics of encompassing and budding of EVs is an active and selective process, and the content of EVs might inform tissue-specificity.

Bioinformatics can contribute substantially to overcoming the above-mentioned challenges, mainly by developing bioinformatic analytic pipeline, which can decode with diverse types of biological data the cell origin, abundance of different types of liquid biopsies, the relative composition of distinct molecules within EVs and so on, and the biological implications conveyed. With such information obtained, it is possible to disclose the genomic and metabolic status of tumor cells in a background of many other cell types, and further to decipher the interactions between tumor cells and other cell types. Other expected bioinformatic input might be integrating information of multianalytes from single samples and then to establish qualitative and quantitative correlation between the disease phenotypes and the molecular profiles of liquid biopsy pool.

Improving the performance of liquid biopsy for PDA is particularly meaningful, however, it is also particularly challenging as circulome biology is relatively underdeveloped in PDA. In the specific context of the PDA, several questions must be addressed with extra caution in future, including: (1) how different is genomic information between primary PDA and liquid biopsies? (2) what genomic and biomolecular alterations detected by liquid biopsies could be directly translated to clinical care? (3) is molecular profiles determined by studying liquid biopsies superior to the conventional histopathology in diagnosing and stratifying PDA patients? (4) is implementing circulomic signatures into the clinical practice labor- and cost-feasible? Carrying out longitudinal monitoring programs and prospective large-scale clinical trials with long term follow-up is essential for addressing these questions.

## Conclusions

Despite recent exciting progresses, clinical application of liquid biopsies remains challenging. In addition to technical limitations, bountiful questions related to the biology of liquid biopsies must be answered, including the origin of liquid biopsies. Comprehensively verifying the diagnostic validity, accuracy, and utility of liquid biopsies as clinical biomarkers will be essential prior to wide adoption of these tests in the clinic.

## Data Availability

N/A, this is a literature review and no data and material are involved.

## Abbreviations

PDA: Pancreatic ductal adenocarcinoma
CT: Contrast-enhanced computed tomography
MRI: Magnetic resonance imaging
EUS: Endoscopic ultrasonography
ctDNA: Circulating tumor DNA
CTCs: Circulating tumor cells
GPC1: Proteoglycan glypican 1
PSA: Prostate-specific an-tigen
CA19-9: Carbohydrate antigen 19-9
MALDI-TOF MS: Matrix-assisted laser desorp-tion/ionization time-of-flight mass spectrometry
TIMP1: Tissue metalloproteinase inhibitor-1
LRG1: Leucine-rich alpha-2-glycoprotein 1
THBS2: Thrombospondin-2
EGFR: Epidermal growth factor receptor
NSCLC: Non-small cell lung cancer
Evs: Extracellular vesicles
UMI: Unique molecular identifier
nDM: New-onset diabetes mellitus
